# Reply: Adjuvant immunochemotherapy with oral Tegafur/Uracil plus PSK in patients with stage II or III colorectal cancer

**DOI:** 10.1038/sj.bjc.6602101

**Published:** 2004-08-10

**Authors:** S Ohwada, Y Morishita

**Affiliations:** 1 Department of Surgery, Gunma University Graduate School of Medicine, 3-39-15, Showa-Machi, Maebashi, Gunma 371-8511, Japan

**Sir**,

We thank Dr C Alliot for his comments and critique in the editorial accompanying our article (Ohwada *et al*, 2004). Regarding the choice of the control arm, we agree that the standard adjuvant treatment for stage III colon cancer since 1990 has been 5-fluorouracil plus leucovorin (5-FU/LV) (NIH, 1990; IMPACT, 1995; Wolmark *et al*, 1999). Now, FOLFOX has become a standard regimen for stage II or III colorectal cancer (Andre *et al*, 2004). Nevertheless, the Ministry of Health and Welfare of Japan did not approve LV for colorectal cancer until June 16, 1999. This study was conducted between October 1994 and March 1997, 2 years before official permission. Therefore, LV was unavailable as a randomised control. As you indicated, there is currently no evidence that UFT is superior to the standard regimen, even when modulated by LV. In a large phase III trial that compared UFT/LV with 5-FU/LV for untreated metastatic colorectal cancer, UFT/LV was found to be a safer, more convenient oral alternative to a standard bolus IV 5-FU/LV regimen, while producing equivalent survival; however, it was associated with an inferior time to disease progression and 22% increase in the risk of disease progression (Douillard *et al*, 2002). Recently, the efficacy of UFT has been determined. In randomised, controlled trials, adjuvant chemotherapy with UFT alone improved the survival of patients with completely resected pathological stage III rectal cancer (Akatsu *et al*, 2004) and stage I adenocarcinoma of the lung (Kato *et al*, 2004), compared with surgery alone.

Dr Alliot was concerned with the high proportion of patients with rectal cancer in the control arm, the impact of the quality of surgery, and the fact that no preoperative radiotherapy was administered. The 5-year disease-free survival for rectal cancer was 69.4% (95% CI: 56.5–82.3%) with PSK and 52.9% (95% CI: 36.2–69.7%) in the controls (*P*=0.133). The difference was not significant, but the high proportion of patients with rectal cancer in the control arm may have pushed the survival for all the patients downward. Therefore, we reanalysed the 5-year disease-free survival adjusted for histologic type and tumour location and found that the survival remained significantly better for the PSK group (stratified logrank; *P*=0.031). The result suggests that the high proportion of patients with rectal cancer did not affect the survival significantly.

As Dr Alliot indicated, the quality of surgery is an important point when conducting any randomised, controlled trial in a surgical field. The recognition that tumour cell involvement in the circumferential margin is important in local recurrence has led to the general use of total mesorectal excision (MacFarlane *et al*, 1993; Kapiteijn *et al*, 2001), in which the entire mesorectum is enveloped and resected using a precise, sharp dissection. Therefore, suitably qualified chief surgeons were trained in the Second Department of Surgery, Gunma University Hospital, in order to standardise surgical quality. We applied total mesorectal resection for Rb or Rab tumours, and tumour-specific mesorectal resection for Ra tumours (at least 4 cm of the anal mesorectum was dissected). Lateral node dissection was performed, while groin dissection was not added. In our study of rectal cancer, the rate of local recurrence was 4.8% in all patients, 2.9% in the control, and 6.1% in the PSK group. The low rate of local recurrence in our study with no preoperative radiation therapy (4.8% at 5 years) is similar to the excellent results achieved using preoperative radiation therapy and standard resection (Kapiteijn *et al*, 2001).

Oncologists agree that an early start for chemotherapy is rationally correct, because surgery provokes the circulation of neoplastic cells (Yamaguchi *et al*, 2000), angiogenesis, and, potentially, the development of micrometastasis, as Dr Alliot indicated. In his editorial, Dr Alliot does not focus on the superior results in the PSK arm, but emphasises the poor results in the control arm. The benefits of an early start for chemotherapy are supported by the significantly superior disease-free survival in the PSK arm. The survival of UFT/MMC in pathologic stage III patients was poor and was similar to that of untreated controls in standard regimens (Francini *et al*, 1994; IMPACT, 1995). This may be explained by the difference in the dose intensity of UFT. The UFT doses used in positive randomised trials include 600 mg body day^−1^ (Malik *et al*, 1990), 300 or 350 mg m^−2^ day^−1^ with LV (Douillard *et al*, 2002), 400 mg m^−2^ for 5 of 7 days (Akatsu *et al*, 2004), and 250 mg m^−2^ day^−1^ (Kato *et al*, 2004), which are all higher than the 300 mg body^−1^ used in our study, despite the lack of LV modulation. The development of more effective agents or regimens should prove that an early start with chemotherapy benefits disease-free survival.

Invoking recently published data from randomised, controlled trials, the survival with PSK/UFT treatment is comparable or superior to standard 5-FU/LV regimens. The 5-year disease-free and overall survival rates were 73.0 and 81.8% for the PSK group, respectively, while the best 5-year disease-free and overall survival rates were 65 and 76%, respectively, in NASBP C-04 (Wolmark *et al*, 1999), and 63 and 70%, respectively, in Intergroup-0089-46-51 (O'Connell *et al*, 1998). When limited to stage III patients, the 4-year disease-free and overall survival rates were 69.1 and 78.2% in the PSK group *vs* 65.3–72.8% for the latter (Porschen *et al*, 2001). Further, in the MOSAIC trial (Andre *et al*, 2004), the 3-year disease-free survival rates of stage II or III patients were 72.9% for 5FM/LV and 78.2% for FOLFOX, while we achieved 77.8% for PSK/UFT. When limited to stage III, the 3-year disease-free survival rates were 65.3% for 5FM/LV and 72.2% for FOLFOX, while we obtained 70.9% for PSK/UFT.

I agree that disease-free survival is more meaningful than overall survival, at least in the case of adjuvant chemotherapy for colorectal cancer (Elfenbein, 2003; Andre *et al*, 2004); second-line therapies for recurrence, including chemotherapy and salvage surgery, can prolong the survival of colorectal cancer patients (de Gramont *et al*, 2000; Saltz *et al*, 2000; Choti *et al*, 2002).

There is no consensus on the optimal duration for adjuvant chemotherapy after colorectal cancer. Currently, a standard adjuvant treatment for stage III colon cancer, is 5-FU/LV for 6–12 months (Francini *et al*, 1994; O'Connell *et al*, 1998; IMPACTB2, 1999; Wolmark *et al*, 1999; QUASAR, 2000; Andre *et al*, 2004). Recurrences generally occur within 2 years after surgery. Indeed, in our study, 71% of cancers recurred during the first 2 years after surgery, and 85% recurred within 2½ years. The mean time to recurrence was 1.9±1.4 years in the PSK group and 1.6±1.1 years in the control group (*P*=0.585). Porschen *et al* (2001) reported that the median time to relapse was 15 months with 5FU/LV treatment and 12 months with 5FU/levamisole treatment. Fluorouracil is a time-dependent agent, and a daily regimen of UFT is an effective way to maintain the blood fluorouracil level. Therefore, the daily, long-term administration of UFT may be beneficial. In addition, oral use of adjuvant chemotherapy with PSK and UFT is less toxic and less complex, as it avoids frequent treatment-related visits and thereby allows patients to receive long-term treatment.

PSK has a wide range of biological activity, remarkable immune-enhancing activity, and a broad antineoplastic scope (Wasser, 2002). Therefore, therapy with PSK differs from therapy with cytokines, such as interferon (IFN) and interleukin (IL)-2. PSK activates NK cells independent of IFN and the IL-2/IL-2 receptor system, and activates lymphokine-activated killer (LAK) cells (Ebina and Murata, 1992; Algarra *et al*, 1997; Harada *et al*, 1997; Pedrinaci *et al*, 1999; Garcia-Lora *et al*, 2001; Garcia-Lora *et al*, 2003). PSK also functions as a specific biochemical modulator of antitumour agents, such as mitomycin C, 5-fluorouracil, cyclophosphamide, bleomycin, CPT-11, cisplatin, and docetaxel (Zhang *et al*, 2003). PSK upregulates the IL-1, IL-6, and IL-8 genes in peripheral mononuclear cells (Hirose *et al*, 1990), as well as the genes for TNF and macrophage chemotactic factors in tumour cells (Ebina and Murata, 1992), and induces apoptosis (Yefenof *et al*, 1995; Zhang *et al*, 2003). In addition, PSK induces differentiation-related genes and produces leukemic cell differentiation *in vitro* (Yefenof *et al*, 1995). Furthermore, PSK suppresses tumour cell invasiveness *via* the downregulation of several invasion-related factors, which include TGF-*β*_1_, urokinase plasminogen activator, and the matrix metalloproteinase (MMP)-2 and -9 (Zhang *et al*, 2000). These activities of PSK are varied and differ from those of levamisole. Although the efficacy of levamisole in a randomised, controlled trial was questionable (QUASAR, 2000), the results for levamisole did not represent those for PSK.

Finally, I thank Dr C Alliot for his comments again. This study shows that PSK is a good candidate for convenient therapy with less toxicity, better compliance, and comparable survival to FOLFOX or 5FU/LV regimens, although our results are not definitive.
